# Melatonin influences proliferation and differentiation of rat dental papilla cells in vitro and dentine formation in vivo by altering mitochondrial activity

**DOI:** 10.1111/jpi.12002

**Published:** 2012-09-04

**Authors:** Jie Liu, Hongyu Zhou, Wenguo Fan, Weiguo Dong, Shenli Fu, Hongwen He, Fang Huang

**Affiliations:** 1 Department of pediatric dentistry, Guanghua School of Stomatology, Hospital of Stomatology, Sun Yat-sen UniversityGuangzhou, China; 2 Department of Oral Anatomy and Physiology, Guanghua School of Stomatology, Hospital of Stomatology, Sun Yat-sen UniversityGuangzhou, China; 3 Guangdong Provincial Key Laboratory of StomatologyGuangzhou, China

**Keywords:** dental papilla cell, differentiation, melatonin, mitochondria, proliferation

## Abstract

Melatonin mediates a variety of biological processes ranging from the control of circadian rhythms to immune regulation. Melatonin also influences bone formation and osteointegration of dental implants. However, the effects of melatonin on dentine formation have not been examined. This study investigated the effects of melatonin on the proliferation and differentiation of rat dental papilla cells (*r*DPCs) in vitro and dentine formation in vivo. We found that melatonin (0, 10^−12^, 10^−10^,10^−8^ m) induced a dose-dependent reduction in *r*DPCs proliferation, increased alkaline phosphatase (ALP) activity, the expression of dentine sialoprotein (DSP), and mineralized matrix formation in vitro. In vivo melatonin (50 mg/kg, BW, i.p.) inhibited dentine formation. Melatonin (10^−8^ m) suppressed the activity of complex I and IV in the basal medium (OS−) and enhanced the activity of complex I and complex IV in osteogenic medium (OS+). These results demonstrate that melatonin suppresses the proliferation and promotes differentiation of *r*DPCs, the mechanisms of which may be related to activity of mitochondrial complex I and complex IV.

## Introduction

Melatonin is produced and secreted by the pineal gland primarily during the night [1–3]. It has a variety of physiological actions including influences on circadian rhythms [3–5], regulation of hormone secretion [6, 7], control of reproduction [8–10], etc. Recently, the investigation and applications of melatonin in the oral cavity has received attention [11–14]. Melatonin has been investigated relative to periodontal disease [11, 15], oral cancer [16], osteointegration of dental implants [17, 18, 19], and inflammatory lesions of the oral cavity [11].

The secretion of melatonin is controlled by the suprachiasmatic nucleus (SCN), which is the endogenous circadian clock located in the hypothalamus [2]. Many studies also reported that tooth development exhibits circadian rhythmicity [20, 21]. Periodic growth incremental lines are found universally in the dental tissues of animals, especially in the dentin and enamel, which reflect circadian rhythms of tooth growth [20, 21]. A previous study had also demonstrated that SCN plays an important role in generating circadian dentine increments [22]. Complete lesion of the SCN led to a failure of dentine increments appearance, and the authors presumed that this was associated with changes in hormones under tight circadian control [22]. Melatonin exhibits circadian rhythmicity, and it influences the circadian fluctuations of some other hormones [9, 23]. Therefore, we presumed that melatonin may be involved in the development of circadian dentine formation.

Dentine formation is similar to bone formation in many ways. Many bone marker proteins such as alkaline phosphatase (ALP) and osteopontin are also markers of the odontoblast [24, 25]. Recent studies have shown that melatonin promotes osteoblast maturation and enhances bone formation by increasing gene expression of many bone marker proteins including bone sialoprotein (BSP), ALP, and osteopontin [18, 26–28]. However, the effect of melatonin on dentine formation is still not clear.

Dental papilla cells are mesenchymal cells, and they have the capability of odontoblastic differentiation [29–31]. In this study, we examined the effects of melatonin on the proliferation and differentiation of the rat dental papilla cells (*r*DPCs) derived from the first mandibular molar dental papilla of 1-day-postnatal Sprague-Dawley (SD) rat using in vitro culture systems. To determine whether melatonin influenced dentine formation in vivo, melatonin was injected into 1-day-postnatal SD rats, and new dentine formation of incisors was examined by fluorescence labeling. In addition, we also investigated the associated mechanisms of melatonin on *r*DPCs' proliferation and differentiation.

The mechanisms of melatonin mediated osteoblast proliferation and differentiation have been documented as an indirect effect via the activation of melatonin receptor MT2 and relevant signal pathways MEK/ERK (1/2) [32, 33]. Meanwhile, mitochondria, the powerhouse of the cell, have the highest intracellular melatonin concentrations. Many effects of melatonin administration may depend on its effect on mitochondrial physiology [34]. So we investigated the associated mechanisms of melatonin on *r*DPCs' proliferation and differentiation by using melatonin receptor antagonist, luzindole, and measuring the activity of mitochondrial complexes (I, II, III, IV).

## Materials and methods

### Cell culture and purification

Dental papilla cells (DPCs) capable of differentiating into odontoblasts were used to assess melatonin's effect on odontoblast differentiation. The *r*DPCs were passaged from the primary generation after isolated from the first molar dental papilla of 1-day-postnatal SD rat (derived from Sun Yat-sen University, Guangzhou, China). Briefly, the dental papilla tissues were carefully isolated using a dental explorer under the stereomicroscope (Stemi2000, Zeiss, Jena, Germany), minced into about 1 × 1 mm^3^ pieces, plated on 25-cm^2^ flask, and incubated in maintenance media with 10% fetal bovine serum (FBS, GIBCO, New York, NY, USA), penicillin (100 U/mL), and streptomycin (100 ug/mL) in incubator equilibrated with 95% air/5% CO_2_ /90% humidity at 37°C. The media were renewed every 3 days. Cells were passaged at 80% confluence and purified using the difference digest method. Passages 3∼4 were used for the following research. The *r*DPCs were identified by keratin and vimentin using immunocytochemistry. *r*DPCs were induced in an osteogenic medium to identify their capability of differentiating into odontoblasts.

### MTT assay

To assess whether melatonin affect *r*DPCs' proliferation, cellular proliferation was assessed using 3-[4, 5-dimethylthiazol-2-yl]-2,5diphenyltertrazolium bromide and thiazolyl blue (MTT; Sigma, St. Louis, MO, USA) assay. The *r*DPCs were plated in 96-multiwell plates at a density of 5 × 10^3^ cells/well and cultured for 24 hr in maintenance media containing 10% FBS. Thereafter, the cells were incubated in the same media containing 2% FBS for 24 hr and subsequently cells were treated for 24, 48, 72, and 96 hr with melatonin as follows: (i) no treatment, (ii) 10^−12^m melatonin, (iii) 10^−10^m melatonin, (iv) 10^−8^ m melatonin, and (v) 10^−10^ m melatonin + luzindole (2 μmol). The incubations were performed in darkness. After incubation, 10 μL MTT (5 mg/mL) was added to each well, the cells were incubated for 4 hr, and then the medium was removed and replaced with 150 μL dimethyl sulfoxide. Absorbance was measured at 490 nm wavelength in an ELISA plate reader (Tecan, Grödig, Austria). Samples were assayed with four replicated samples in each group at the same time, and the mean optical density (OD) values were measured. The experiment was repeated at least three times.

### ALPase activity assay

To assess whether melatonin modulates ALP activity, a marker of odontoblast differentiation, ALP levels were measured by analyzing the rate of p-nitrophenyl phosphate disodium hexahydrate (pNPP) hydrolysis. Briefly, *r*DPCs were plated in 96-multiwell plates at a density of 5 × 10^3^ cells/well and cultured until they reached confluence. Then the cells were treated with the following agents for 3 days: (i) OS−M−, (ii) OS−M+ (10^−8^ m melatonin), (iii) OS + M−, (iv) OS + M+ (10^−8^ m melatonin), and (v) OS + M + (10^−8^ m melatonin) + luzindole (2 μmol) (OS: osteogenic medium, containing 50 μg/mL ascorbate, 100 nm dexamethasone, 2 mm β-glycerophosphate). The pNPP product was generated using a p-nitrophenol phosphate stock (Jiancheng, Nanjing, China) and analyzed according to the manufacturer's instructions. Briefly, after washing three times with PBS, the cells were added 50 μL 0.1% TritonX-100 per well and incubated at 4°C overnight. Then, the pNPP reaction mixture was added to the plates and incubated in 37°C for 30 min. The reaction was terminated with 1.0 m NaOH and immediately analyzed at 520 nm by ELISA plate reader (Tecan, Grodig, Austria). Samples were assayed with four replicated samples in each group at the same time, and the mean OD values were analyzed. All the measurements were performed in triplicate.

### Dentine sialoprotein assay

To assess whether melatonin modulate DSP, a specific marker of odontoblast differentiation was evaluated by immunocytochemistry. The *r*DPCs were seeded at a cell density 1 × 10^4^ cells per well in 48-well culture plates, incubated in maintenance media for 1 day, and then the cells were exposed to the following conditions for 7 days: a) OS−M−, (b) OS−M+ (10^−8^ m melatonin), c) OS + M−, and d) OS + M + (10^−8^ m melatonin). After incubation in the condition media, the cultures were fixed in 10% neutral formalin buffer. Endogenous peroxidase was blocked with 3% H_2_O_2_. Following treatment with 10% bovine serum albumin, cells were incubated with goat anti-DSP polyclonal antibody (Santa Cruze, CA, USA) using 1:50 dilution in PBS containing 1% bovine serum albumin overnight at 4°C and kept for 1 hr at room temperature. Thereafter, the cells were incubated with rabbit anti-goat polyvalent antibody (Zymed Santiago, USA).After incubation in streptavidin peroxidase (Zymed Santiago), the immunoreactive product was visualized after incubation with diamino benzindine (DAB). All dilutions and washes between procedures were performed using PBS unless otherwise specified. For immunohistochemical controls, primary antibody was replaced with PBS. The images were obtained by inverted phase contrast microscope Axiovert 40(Zeiss, Gottinge, Germany), and image analysis was performed by image analysis software Image-pro plus 6.0 (Media Cybernetics, Bethesda, MD, USA).

### Alizarin red staining

The effect of melatonin on the formation of mineralized nodules was examined by alizarin red S staining. The *r*DPCs were seeded at a cell density of 2 × 10^4^ cells per well to 12-well culture plates and cultured in growth medium until they reached confluence. At confluence, the medium was changed to condition medium. After 21 days, the cells were fixed with 10% neutral formalin buffer for 15 min and stained with 0.1% alizarin red S solution. To determine the degree of mineralization, alizarin red S bound to cultures was extracted by incubation with 250 μL of 1% hydrochloric acid in 70% ethanol [27]. The absorbance was measured at 450 nm wave in an ELISA plate reader (Tecan).

### Isolation of mitochondria

The *r*DPCs were seeded to 75-mL flask and incubated in maintenance media for 1 day and then the cells were exposed to the following conditions for 3 days: OS−M− and OS−M+ (10^−8^ m melatonin) or OS + M− and OS + M+ (10^−8^ m melatonin). Mitochondria were isolated from the cultured *r*DPCs as our pervious study described [35]. Briefly, the *r*DPCs (3 × 10^7^) were harvested by centrifugation at 600 g for 10 min at 4°C. The cell pellets were washed once in PBS and then resuspended in three volumes of isolation buffer (pH 7.4). The buffer solution consisted of HEPES (20 mm), KCl (10 mm), MgCl_2_ (1.5 mm), EGTA (1 mm), EDTA (1 mm), dithiothreitol (1 mm), phenylmethylsulfonyl fluoride (10 mm), aprotinin (10 μm), leupeptin (10 μm), and sucrose (250 mm). After chilling on ice for 3 min, the *r*DPCs were disrupted by 40 strokes of a glass homogenizer. The homogenate was centrifuged twice at 2500 g at 4°C to remove unbroken cells and nuclei. Then the mitochondria were pelleted by centrifugation at 12,000 g for 30 min. The mitochondrial pellet was resuspended in isolation buffer. Protein contents were determined using the method of Bradford. All the procedures were carried out at 4°C or on ice.

### Measurement of mitochondrial complex activity

The measurement of the specific activity of the respiratory chain complexes was performed as described [35]. All assays were performed in 1 mL final volume with 5–10 μg mitochondrial proteins, and the linear change in absorbance was measured for 3 min. All concentrations below are final concentrations.

Complex I (NADH ubiquinone oxidoreductase): the reaction mixture consisted of 10 mm Tris–HCl pH 8.0 buffer, 80 μm 2, 3-dimethoxy-5-methyl-6-decyl-1, 4-Benzoquinine (DB), 1 mg/mL BSA, 0.25 mm KCN, and 0.4 μm antimycin. After incubating mitochondria in the reaction mixture at 30°C for 5 min, oxidation of NADH (200 μm) was monitored at 340 nm (*є*5.5/mm per cm).

Complex II (succinate ubiquinone oxidoreductase): mitochondria were incubated in 50 mm potassium phosphate buffer pH 7.4 containing 20 μm succinate. After addition of assay mixture consisting of 50 μm 2, 6-dichlorophenolindophenol (DCPIP), 2 μg/mL rotenone, 2 mm KCN, and 2 μg/mL antimycin, 25 μm DB was mixed. The reduction of DCPIP in association with CII-catalyzed DB reduction was measured at 600 nm (*є* 19.1/mm per cm).

Complex III (ubiquinol cytochrome c oxidoreductase): mitochondria were suspended in 50 mm Tris–HCl buffer (pH 7.4), containing 1 mm EDTA, 250 mm sucrose, 2 mm KCN, and 50 μm oxidized cytochrome c. After the addition of 80 μm reduced DB (DBH2), reduction in cytochrome c was measured at 550 nm (*є* 19.0/mm per cm).

Complex IV (cytochrome c oxidase): mitochondria (2 μg protein) were permeabilized in 10 mm Tris–HCl (pH 7.0), 25 mm sucrose, 120 mm KCl, and 0.025% n-dodecyl-b-dmaltoside, and 50 μm reduced cytochrome c was added. The oxidation of cytochrome c was measured at 550 nm (*є* 19.0/mm per cm).

### Analysis of effect of melatonin on dentin formation

The following experiments were approved by the Animal Care and Use Committee of the Sun Yat-sen University. Twelve 1-day-postnatal SD rats coming from the same nest were divided into two groups according to the random principle. Experimental rats received a melatonin injection (50 mg/kg, BW, i.p.) every day for 19 days. Control rats were injected with physiological saline. To measure the volume of newly formed dentin of the incisor, rats received tetracycline injections (25 mg/kg, BW, i.p.) on days 3, 9, and 15 and calcein injections (10 mg/kg, BW, i.p.) on days 6, 12, and 18. On day 20, rats were deeply anesthetized with 10% chloral hydrate (0.35 mL/100 g BW, i.p.) and transvascularly perfused with 4% paraformaldehyde in 0.1 m PBS(pH 7.4). The incisors were excised and further fixed in 10% phosphate-buffered formalin. Then longitudinal sections were cut at 30 μm and observed with the fluorescence microscope. The width between the fluorescence-labeled lines with tetracycline and calcein were calculated as the newly formed dentine using image software (Axiovision. Ac Rel.4.5, Zeiss, Gottinge, Germany).

### Statistical analysis

Results are listed as the mean ± S.E.M. Data were analyzed by means of analysis of variance (ANOVA). The Student-Newman–Keuls test was used when significant overall F values were obtained. *P* value <0.05 was considered to be significant.

## Results

**Fig. 1:**
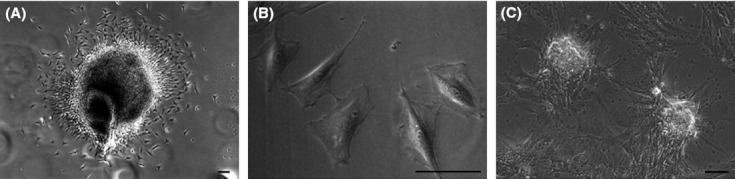
Rat dental papilla cells (DPCs) ′ culture and purification. (A) DPCs' primary culture: cells were migrated from tissue pieces after 2 days culture. (B) DPCs' purification: cells were homogenous, large, polygonal and had abundance cytoplasm. (C) Mineralized matrix secreted by DPCs after osteogenic inducing 21 days. Scale bars = 100 μm.

The *r*DPCs were isolated from first mandibular molar of 1-day-postanal SD rat. When small pieces of dental papilla were cultured in 25-mL flask, *r*DPCs migrated from the pieces at the days 2–3 (Fig. 1A) without any treatment and proliferated rapidly. *r*DPCs were homogenous, large, polygonal fibroblastic cells and had abundance cytoplasm and centrally localized single nuclei (Fig. 1B). *r*DPCs were positive for antivimentin staining and negative for antikeratin. When the cells were induced by osteognic medium for 21 days, the mineralization nodules can be observed (Fig. 1C). All of these indicated that the cells obtained from our experiments are mesenchymal types and have the capability of odontoblast differentiation.

**Fig. 2:**
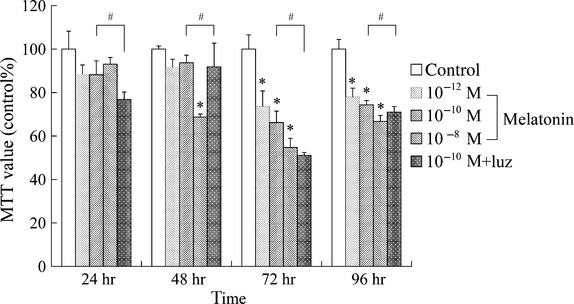
Effect of melatonin on the proliferation of rat dental papilla cells (DPCs). Cells were seeded in 96-multiwell plates at a density of 5 × 103 cells/well, and cultured for 24 hr in maintain media containing 2% fetal bovine serum. Subsequently cells were treated for 24, 48, 72 and 96 hr with the different concentrations melatonin: (0; 10–12; 10–10; 10–8 m) or melatonin(10–10 m)/luzindole(2 μmol). The cells viability was measured by MTT method. Melatonin inhibited the proliferation of DPCs in a dose-dependent manner (*P<0.05). Luzindole could not prevented melatonin-induced decreasing of cells proliferation(#*P* > 0.05).

To estimate the effects of melatonin on proliferation of *r*DPCs, cell activity was examined by MTT assay. Fig. 2 displays the activity of cells after incubation with various concentrations of melatonin. When cells were exposed to melatonin for 48 hr, high concentration group (10^−8^ m) showed the inhibitory effect. After 72-hr and 96-hr incubation, the inhibitory effect became more obvious and was in a dose-dependent manner. However, the melatonin receptor antagonist luzindole could not counteract melatonin-induced inhibition effects.

**Fig. 3:**
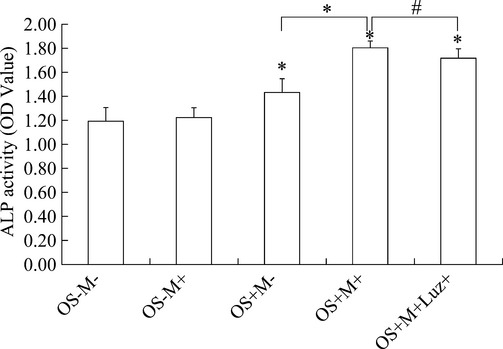
Alkaline phosphatase activity was evaluated by the PNPP method. Cells were plated in 96-multiwell plates at a density of 5 × 103 cells/well and cultured until they reached confluence. Then the medium were changed to the condition medium for 3 days. Melatonin alone (OS−M +) could not increase alkaline phosphatase (ALP) activity, but it increased ALP activity in the osteogenic medium (OS + M +). (*P < 0.05). Luzindole could not prevented melatonin-induced increasing of ALP activity (#*P >* 0.05).

**Fig. 4:**
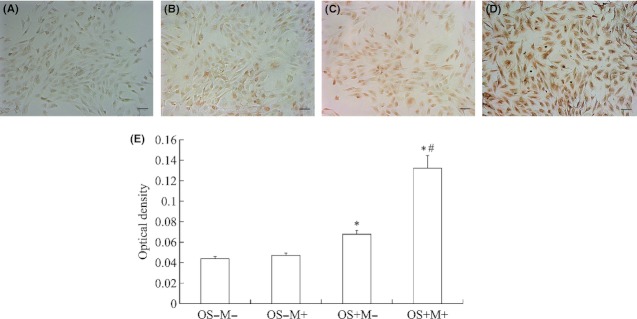
Dentine sialoprotein expression was examined by immunocytochemistry. Cells were seeded in 48-well culture plates at a cell density 1 × 104 cells per well and incubated in maintain media for 1 day, then the medium were changed to the condition medium for 7 days. Then the cells were stained by immunocytochemistry method (A: OS−M−, B: OS−M +, C: OS + M−, D: OS + M+, Scale bars = 100μm). The optical density was examined by image software IPP.6.0. (E, *P<0.05, compared to OS−M−, #*P*<0.05, compared to OS + M−).

**Fig. 5:**
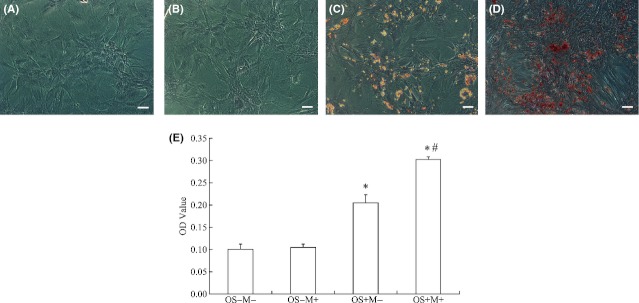
Mineralized nodules were examined by Alizarin red S staining. dental papilla cells were incubated with the condition medium (A: OS−M−,B:OS−M +, C:OS + M−, D:OS + M +) for 21days, then the Mineralized nodules were stained by Alizarin red S and Alizarin red S bound to cultures was extracted by incubation with 250 μL of 1% hydrochloric acid in 70% ethanol. The absorbance was measured at 450 nm wave in an ELISA plate reader. Melatonin stimulated the mineralized matrix formation significantly (E) (*P < 0.05, compared to OS−M−, #*P* < 0.05, compared to OS + M−).Scale bars = 100 μm for A, B, C, D.

To examine the effect of melatonin on the differentiation of *r*DPCs, ALP activity, DSP expression, and mineralization nodules were detected by PNPP method, DSP immunocytochemistry, and alizarin red staining. Fig. 3 presents the effect of melatonin on ALP activity of *r*DPCs. Melatonin supplemented with osteogenic medium (OS + M +) increased ALP activity over that osteogenic medium alone (OS + M−). Moreover, luzindole did not weaken the melatonin-induced ALP activity in osteogenic medium yet. Fig. 4 illustrates the DSP expression after incubation with melatonin alone or supplemented with osteogenic medium for 7 days. Results showed that melatonin alone (OS−M +) did not increase DSP expression (Fig. 4B, E), while osteogenic medium (OS + M−) could increase DSP expression (Fig. 4C), but the color is weaker than the melatonin and osteogenic medium incubation group (OS + M +) (Fig. 4D,E). Fig. 5 shows that melatonin alone did not stimulate the mineralized matrix formation (Fig. 5B,E), but it did enhance the mineralized matrix formation in the osteogenic condition (Fig. 5D,E).

**Fig. 6:**
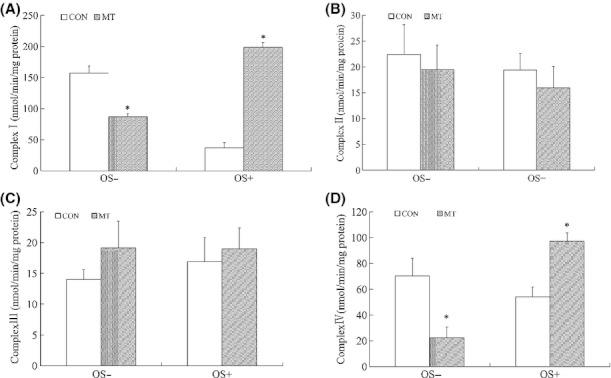
Effects of melatonin on the mitochondrial complexes activity. dental papilla cells were seeded and incubation with or without melatonin in growth medium (OS−) or osteogenic medium (OS+) for 3 days. Then the mitochondrial were isolated and the specific activity of the respiratory chain complexes was measured. Results areexpressed as nmol/min/mg protein for Complex I (A), Complex II (B), Complex III (C), Complex IV (D). (*P < 0.05, compared to control.)

The mechanism of action of melatonin on the proliferation and differentiation of DPCs is still unclear. We measured the mitochondrial complex activity to estimate mitochondrial function. Results showed that when *r*DPCs were incubated with basal medium (OS−), melatonin significantly inhibited the activity of complex I and complex IV (Fig. 6A,D). And that when *r*DPCs were incubated with osteogenic medium (OS+), melatonin promoted the activity of complex I and complex IV (Fig. 6A,D). The changes in complex II and complex III were not obvious (Fig. 6B,C).

**Fig. 7:**
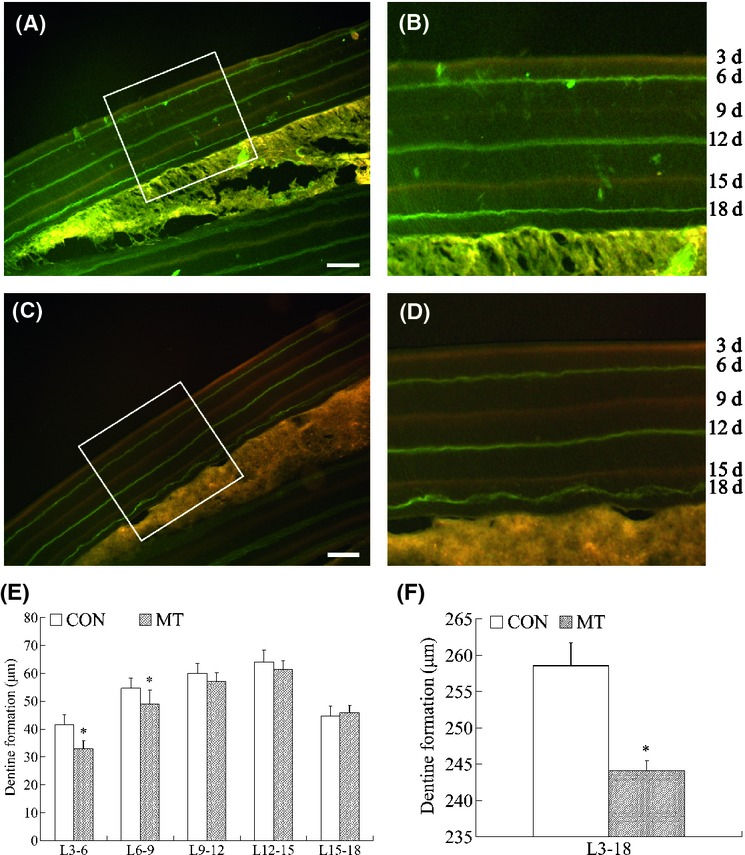
Melatonin effects on rat incisor dentine formation in vivo. 1-day-postnatal SD rats received a melatonin injection or physiological saline for 19 days. On days 3, 9 and 15, rats received tetracycline injections; on days 6, 12 and 18, rats received calcein injections. The newly formed dentine was observed with fluorescence microscope (A and B: control, C and D: melatonin group). To evaluate the volume of newly formed dentine, the widths between the line labeled with tetracycline and the line labeled with calcein were measured by image software. The newly formed dentine of melatonin treated group was significant less than the control during days 3–6 and days 6–9 (E) (*P < 0.05). And total of new dentine formation of melatonin treatments were still less than control (F) (*P < 0.05). Scale bars = 100 μm for (A, C).

To evaluate the influence of melatonin on dentine formation in vivo*,* 1-day-postnatal SD rats received melatonin or physiological saline for 19 days. Results showed that melatonin prevented the dentine formation in early stage. The newly formed dentine of melatonin-treated animals was significant less than the control during days 3–6 and days 6–9. After 9 days, the differences in newly formed dentine were not obvious between melatonin treatments and control (Fig. 7A–A–A–A–E). However, total new dentine formation after melatonin treatments was still less than the control (Fig. 7F).

## Discussion

Mounting evidence shows that melatonin modulates the proliferation of a variety of cell types, but the effects of melatonin on cell proliferation are different depending on the cell types, the melatonin concentrations, and the culture conditions [27, 32, 36–39]. Radio et al. [32] reported a subtle significant inhibition of human adult mesenchymal stem cell proliferation occurred following incubation with melatonin alone or osteogenic medium supplemented with 50 nm melatonin. In contrast, Nakade et al. [36] and Satomura, et al. [27] reported that melatonin stimulated proliferation of human osteoblasts. Our prior study showed that melatonin inhibited *r*DPCs' proliferation [40]. In this study, we found that melatonin suppressed the *r*DPCs' proliferation in a dose-dependent manner, even in very low concentration (10^−12^ m). We also demonstrated that exogenous melatonin reduced dentine formation in vivo. This indicated that melatonin may be involved in regulating the proliferation of *r*DPCs in physiological progress.

To estimate the effects of melatonin on the odontogenic differentiation of rDPCs, we investigated ALP activity, DSP, and extracellular matrix mineralization as markers of odontogenic differentiation. ALP is a maker for early odontogenic differentiation. We found that melatonin supplemented with osteogenic medium (OS + M +) increased ALP activity, but melatonin alone (OS−M +) could not enhance ALP activity. This indicated that melatonin may influence ALP activity of differentiated odontoblasts rather than undifferentiated rDPCs. In the past, DSP was known as a specific marker of odontoblast [41]. Recent studies showed that it also expressed in bone [42], cementum [43], and other tissues [44], but the expression of DSP in nondental tissue is much lower than dentin [40]. So, DSP is still considered to be an important marker of the odontoblast. We preliminarily found that melatonin supplemented with osteogenic medium (OS + M+) enhanced the expression of DSP by immunocytochemistry. From the above findings, we surmise that melatonin may participate in the differentiation of *r*DPCs.

The mechanism of action of melatonin on cell proliferation and differentiation may depend on its receptors, which are widely distributed. The binding site of melatonin contains G-protein-coupled melatonin receptors (MT_1_/MT_2_), nuclear receptor (ROR/RZR receptor), calmoduline, and mitochondria [9]. Among them, G-protein-coupled receptors (MT_1_/MT_2_) are the classical pathway of melatonin. In this study, melatonin receptor antagonist luzindole could not inhibit melatonin-induced reduction in cell proliferation and the increase in ALP activity. So we assume that melatonin influences the proliferation and differentiation of rat dental papilla cells, and dentine formation may be not associated with the pathway of G-protein-coupled melatonin receptors (MT_1_/MT_2_).

The melatonin binding sites on mitochondrial membranes have been identified, and the effects of melatonin on mitochondria are definite [9, 34]. Melatonin may time-dependently improve the activity of complex I and complex IV of the respiratory chain after administration to rats. Also, melatonin was shown to increase the activity of complex I and complex IV to prevent the mitochondrial damage induced by ruthenium red in vivo [45]. In this study, we measured the activity of complexes (I, II, III, IV) under different conditions (OS− and OS+) to evaluate the melatonin influence on mitochondria. Results showed that in growth medium (OS− melatonin reduced the activity of complexes I and IV. This may be associated with melatonin's inhibitory effects on *r*DPCs' viability and dentine formation. We presume that melatonin influences the *r*DPCs' proliferation, and dentine formation may be through its inhibition of mitochondrial function. In contrast, in the osteogenic medium (OS+), melatonin increased the activity of complexes I and IV. Melatonin enhanced the ALP activity, DSP expression, and mineralized matrix formation when *r*DPCs were induced by osteogenic medium. It is assumed that melatonin plays a role in the DPCs in the development of the tooth, the mechanisms of which involve activity of mitochondrial complexes I and IV.

To investigate the effect of melatonin on dentine formation in vivo, melatonin was injected into 1-day-postnatal SD rats, and new dentine formation of rat incisors was examined using a histological method. As a result, new dentine formation of rat incisor was significantly decreased by melatonin injection compared with control in the early stage, and after 9 days, new dentine formation was not different between melatonin treatment and control groups. The mechanisms of these effects on dentine formation may be related to the synthesis and secretion of endogenous melatonin and to the degree of *r*DPCs' differentiation. Early studies have shown that the pineal gland of the neonatal rat does not synthesize melatonin because of the low activity of the final enzyme in the synthetic pathway, hydroxyindole-O-methyltransferase (HIOMT). However, very small amounts of melatonin may be derived from maternal milk [5]. Melatonin in the pineal gland and in the blood of neonatal rats is detected at 12 hr after birth, and melatonin continues to increase in proportion to body weight until postnatal 5 days. The rhythmic synthesis of melatonin in the rat pineal gland appears during the second postnatal week [46]. In the present study, we found that exogenous melatonin reduced dentine formation of rat incisor until postnatal day 9, the interval during which endogenous melatonin levels are low. After postnatal day 9, the endogenous melatonin concentrations increased and they become rhythmic; thus, both aspects of dentine formation as observed in this study may be explained on the basis of normal endogenous melatonin formation. On the other hand, the degree of DPCs' differentiation may be another factor. The differentiation of odontoblasts from DPCs is a complex process involving several steps (pre-odontoblasts, polarized odontoblasts, terminally differentiated odontoblasts) [47–49]. In the present study, the incisors of neonatal rats were in the late bell stage, and most cells were pre-odontoblasts. After several days, the amount of functional odontoblasts increased. In the different stages of development of dentine formation, it is possible that the roles of melatonin regulating the cell cycle and differentiation process differ. This will require further investigation.

In summary, the present results demonstrate that melatonin alters the proliferation of *r*DPCs in a dose-dependent manner and prevents early dentin formation in vivo. Moreover, melatonin increases ALP activity, DSP expression, and mineralized matrix formation. These actions may be associated with alterations in the function of the mitochondria. These findings strongly suggested that melatonin may regulate dentine formation and tooth development. Another possibility is that melatonin could mediate the formation of tooth abnormalities, such as macrodontia and microdontia. To elucidate these issues, in vivo studies would be very helpful and should be performed in the future.
